# Supplementary figures and images for: Five decades of change in somatic growth of Pacific hake from Puget Sound and Strait of Georgia
Source: PeerJ. 2022 Jul 13;10:e13577. doi: 10.7717/peerj.13577 (PMC9288167; doi:10.7717/peerj.13577)

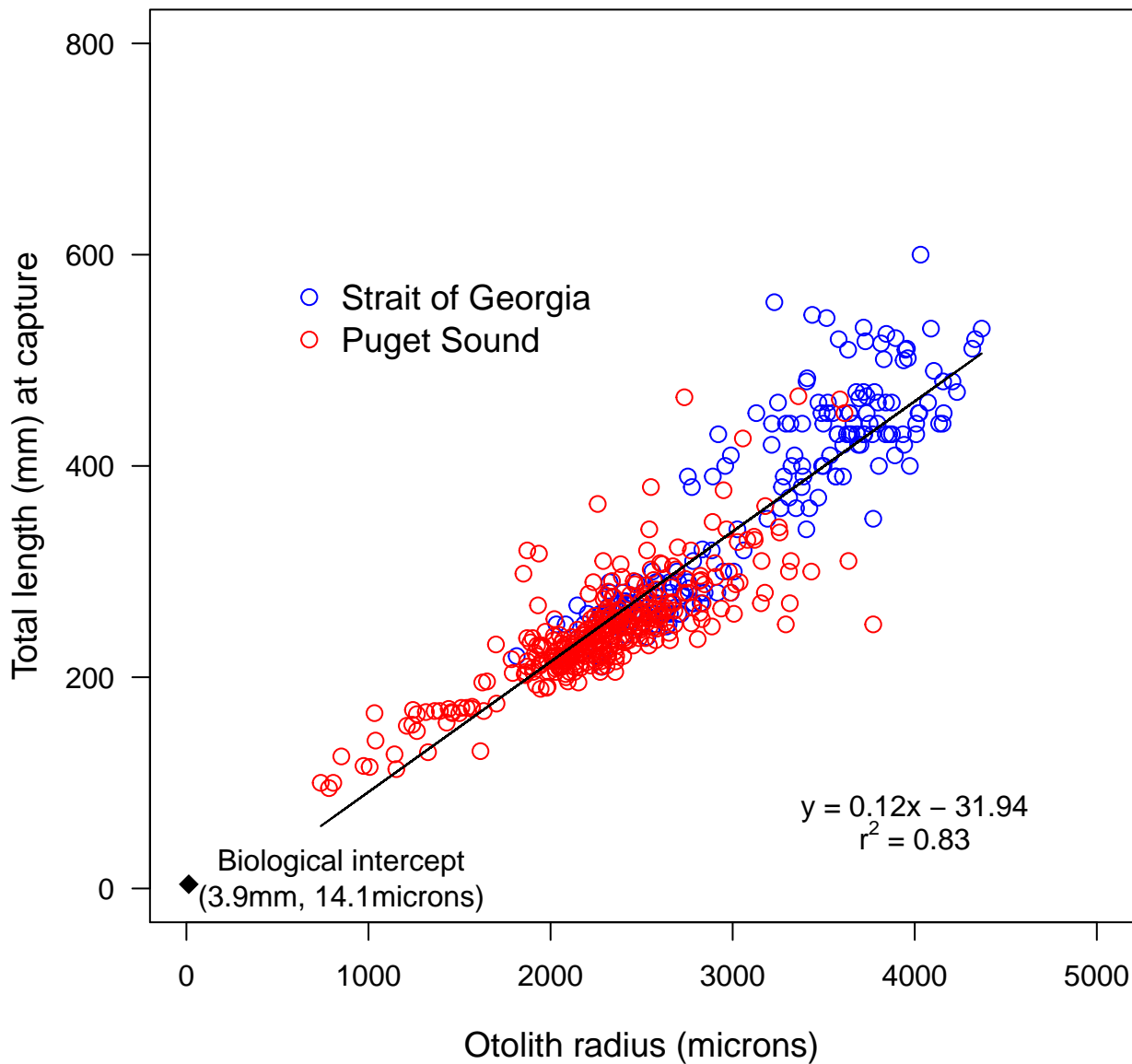

Supplement: Supplemental Information 1 — Biological intercepts of length and otolith radius at first feeding were defined as 3.9 mm and 14.1 µm, respectively [file peerj-10-13577-s001.pdf]
